# Misdiagnosed metabolic bone abnormality: a case report

**DOI:** 10.1186/s13256-023-04164-w

**Published:** 2023-10-20

**Authors:** Mohammed Alsabri, Hannah Street, Aaron Sircy, Bahaaeldin Labib

**Affiliations:** 1 Pediatrics, 1 Brookdale University Hospital and Medical Center, 1Brookdale Plaza, Brooklyn, NY 11212 USA; 2 Emergency Medicine Department, Al Thawra Modern General Hospital (TMGH), Sana’a City, Yemen; 3 NYU Langone Medical Center, 550 1st Avenue, New York, NY 10016 USA; 4 CMEF Aultman Hospital, 2600 6th st. SW, Canton, OH 44710 USA; 5 Rutgers-Robert Wood Johnson University Hospital, 200 Somerset Street, New Brunswick, NJ 08901 USA; 6 College of Osteopathic Medicine, NYIT, Glen Head, NY USA

**Keywords:** Metabolic bone disease, Rare genetic disease, Amelogenesis imperfecta, Enamel hypoplasia, Renal enamel syndrome

## Abstract

**Background:**

Metabolic bone disease causes significant morbidity and mortality, especially when misdiagnosed. With genetic testing, multiple disease pathologies can be analyzed.

**Case presentation:**

A 5-year and 9-month-old otherwise healthy Yemeni girl presented to her Yemen physician for evaluation of inward bending of her right knee and short stature. After extensive medical testing, she was given a diagnosis of hypophosphatemic rickets and growth hormone deficiency and started on treatment. Despite appropriate treatment, however, her condition continued to progress, prompting her family to pursue additional workup including genetic testing outside of Yemen. Genetic testing ultimately revealed a variation of unknown significance associated with amelogenesis imperfecta.

**Conclusions:**

Hypophosphatemic rickets secondary to renal tubular acidosis was the working diagnosis. However, the patient’s condition did not improve. Further genetic testing revealed a variation of unknown significance associated with amelogenesis imperfecta. We aim to present this case, provide an overview of the causes, and diagnostic metabolic bone health evaluation.

## Background

Pediatric bone growth is a dynamic process that occurs throughout childhood and adolescence. The growth and formation of healthy bones rely on the essential elements calcium, phosphorus, 1,25-dihydroxyvitamin D [1,25(OH)2D], and parathyroid hormone [1, 2]. The concentrations are controlled by communication between the parathyroid glands, bone, intestines, and kidney. Improper mineralization of the growth plate is referred to as rickets and presents in children present with variety of complications, including lower extremity bowing, limb pain, failure to thrive, and dental disease [2–5]. The underlying cause of improper bone formation is broad, depending on the essential element found to be deficient and includes a host of metabolic causes, malnutrition, malabsorption, and renal tubular acidosis [6].

Improper bone formation has additionally been found to be associated with defectively formed dental enamel, also referred to as amelogenesis imperfecta (AI). Amelogenesis imperfecta refers to a class of heritable conditions resulting in defective dental enamel formation, presenting with delayed tooth eruption, crown resorption, pulpal calcifications, dental follicular hamartomas, and gingival hypertrophy [7, 8]. Case reports in the literature note an entity referred to as renal enamel syndrome where AI is seen in conjunction with nephrocalcinosis. The first case report from 1972 documented a pair of siblings with AI and severe renal failure secondary to nephrocalcinosis. Subsequent case reports have found similar associations between AI and unexplained nephrocalcinosis with additional features including normal plasma calcium levels, alkaline phosphatase levels, and parathyroid function [9]. Further research seems to suggest a possible contiguous gene syndrome for renal enamel syndrome with studies showing autosomal dominant, autosomal recessive, and x-linked inheritance modalities, although further research into genetic causes of AI and nephrocalcinosis is needed [9].

The pathophysiology of renal enamel syndrome is largely unknown, although two theories have been proposed. One theory suggests an underlying interstitial matrix abnormality causing dystrophic kidney calcification and abnormal tooth enamel production [10]. The second theory proposes that dental proteins previously thought to be tissue specific may also be expressed in nondental tissues; however, the role these proteins may play in renal function requires further study [10].

We present a case study of an 8-year-old Yemen girl who presented to her physician at age 5 years and 9 months for bony changes including slight right knee genu valgum. Misdiagnosed, her condition worsened for multiple years. Eventually, a diagnosis of hypophosphatemia rickets due to renal tubular acidosis was hypothesized. Further genetic testing showed a new homozygous missense variant in a gene that has been associated with amelogenesis imperfecta. We would like to share our unique case experience with the pediatric medical community.

## Case presentation

**Fig. 1:**
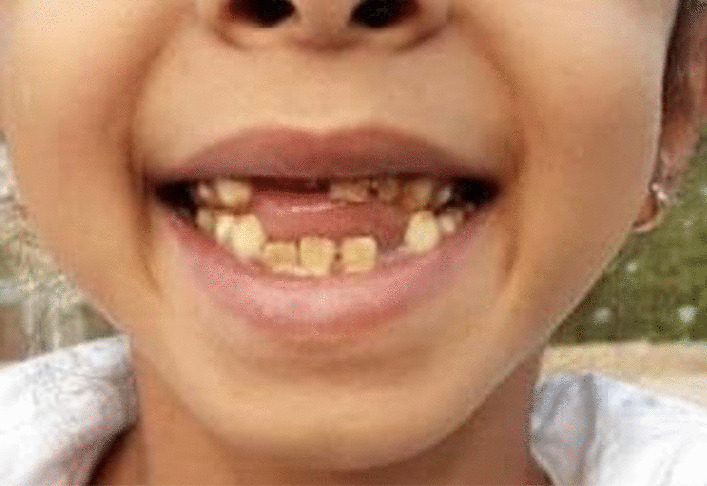
Tooth discoloration and malocclusion

On 24 December 2018, a previously healthy young Yemeni girl aged 5 years and 9 months presented to her physician in Yemen accompanied by her parents for evaluation of inward bending of her right knee. Her parents first noticed the genu valgum of her right knee in 2017, with progressive worsening since then. In addition, parents noticed yellow teeth discoloration and teeth malocclusion as shown in Fig. 1. The child was developmentally normal up until 2017, meeting all developmental milestones appropriately. She had an unremarkable birth history: born via spontaneous vaginal delivery with a normal birth weight and an uncomplicated delivery and neonatal period. All her vaccinations were up to date, and she had no history of feeding difficulties. The family history was unremarkable except for consanguinity of her parents (they are first cousins). Notably, the patient has three sisters and one brother; one older sister of 14 years old who is healthy, and two sisters of 7 and 4 years old, both of them suffering from teeth discolorations, who also look shorter than other average children (based on the parents observation). This is the same as her 9-year-old brother. However, none of the siblings have officially been diagnosed with bone, teeth, or growth abnormalities.

Physical examination revealed a short, frail girl with a height of 95 cm (< 3rd percentile) and a weight of 12 kg (< 3rd percentile). A detailed musculoskeletal exam revealed bilateral genu valgum and frontal bossing. There were no signs of rachitic rosary, widening of the wrists. Upper limb examination was normal. There was no evidence of skin hyperpigmentation, dry scaly skin, or coarse brittle hair. The remainder of the systems examinations were unremarkable.

Laboratory workup was notable for normal calcium level (8.8 mg/dL, 8.8–10.32 mg/dL) elevated alkaline phosphatase (369 U/L, < 269 U/L), and low vitamin D (16.5 ng/L, > 30 ng/L). Complete blood count, antistreptolysin O (ASO), and C-reactive protein (CRP) were normal. A bilateral plain film radiograph of the patient’s knees showed slight genu valgum of both legs and widening of the growth plates. The patient was diagnosed with vitamin D deficiency and started on vitamin D 2000 IU per day, which was later increased based on serum vitamin D levels.

A year later, at the age of six years and nine months, the patient was reevaluated given continued progression of her symptoms despite vitamin D and calcium supplementation. Over the elapsed period, her height only increased by 1 cm, reaching 96 cm. In addition, the child started developing tooth decay and peridental abscesses. Further investigations revealed a normal thyroid panel, low phosphate levels (2.4 mg/d, 3.4-6.2 mg/dL), low growth hormone (GH), and low insulin-like growth factor 1 (ILGF-1). At this point, a diagnosis of growth hormone deficiency was considered, and the patient was started on the growth hormone replacement agent Norditropin at a dose of 0.17 mg/kg/week.

Over the next year, the patient continued Norditropin therapy. After starting growth hormone therapy, she experienced significant worsening of her condition, with complete halting of her vertical growth, continued worsening of her genu valgum, and increased weakness leading to difficulty ambulating. Her parents decided to pursue additional workup in Egypt.

**Fig. 2:**
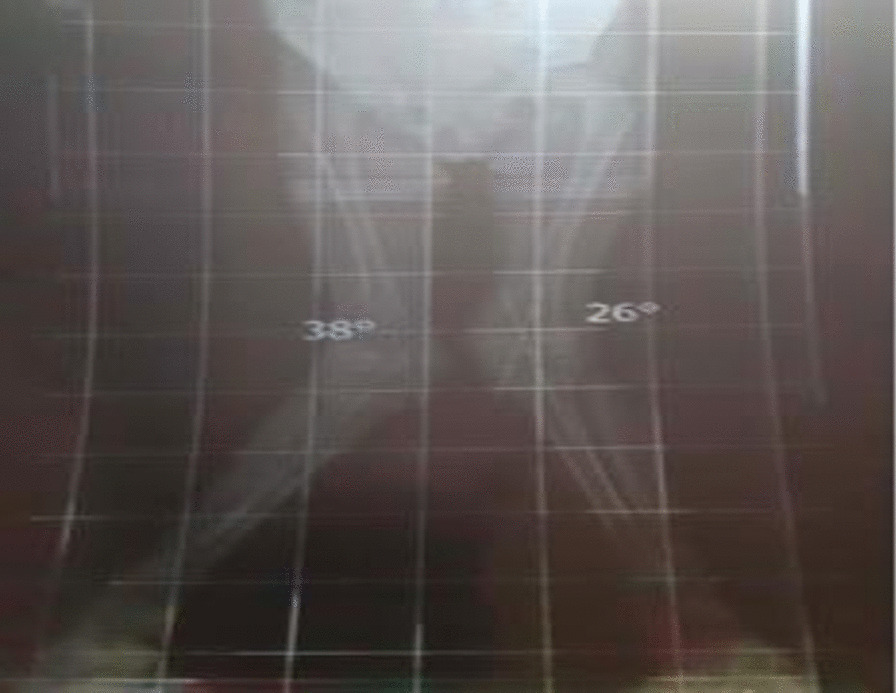
Bilateral lower extremity x-ray showing deterioration of the genu valgum deformity and decreased bone density. Right fibular shaft fissure fracture line without displacement noted with attempts at healing. Normal articulation of joint spaces

On 1 March, 2021, she presented for reevaluation in Egypt. Bilateral lower extremity radiographs showed significant deterioration of the genu valgum compared with her initial x-ray (Fig. 2). This prompted further investigation with a bone scan, which indicated a bone age of 28 months for the now 8-year-old girl. Official bone densitometry was also performed, reporting a total bone mineral density of 0.413 g/cm^2^, total body mineral content is 336.7 g, total bone mineral density of the lumbar spine is 0.316 g/cm^2^ (*Z* score −4). The patient’s bone mineral density readings were compared and plotted against peak bone mineral density readings and bone mineral density readings of age- and sex-matched healthy subjects. A *Z* score of −4.0 is lower than normal limits for the patient age and sex indicating osteoporosis [estimation of the bone mineral density was achieved at the lift hip and lumbar spine (L1–L4) as well as total body using dual-energy x-ray absorptiometry (DEXA) technique].

Repeat labs at that time were significant for decreased serum phosphorus (1.5 mg/dL, 2.5–6.8 mg/dL), mild to moderate vitamin D deficiency (18 ng/L, 10–25 ng/mL) and increased 24-hour urinary phosphate (362 mg/24 hours). Given the abnormal urine studies, concerns for renal involvement led to a renal ultrasound, which was significant for bilateral bulky kidneys with non-homogeneous texture and prominent medullary pyramids, suggesting parenchymal insult. A detailed ophthalmic exam, including slit lamp exam, was performed to evaluate for cystinosis, which was normal. Considering these results, growth hormone treatment was suspended, and phosphate and vitamin D supplements were restarted.

**Fig. 3:**
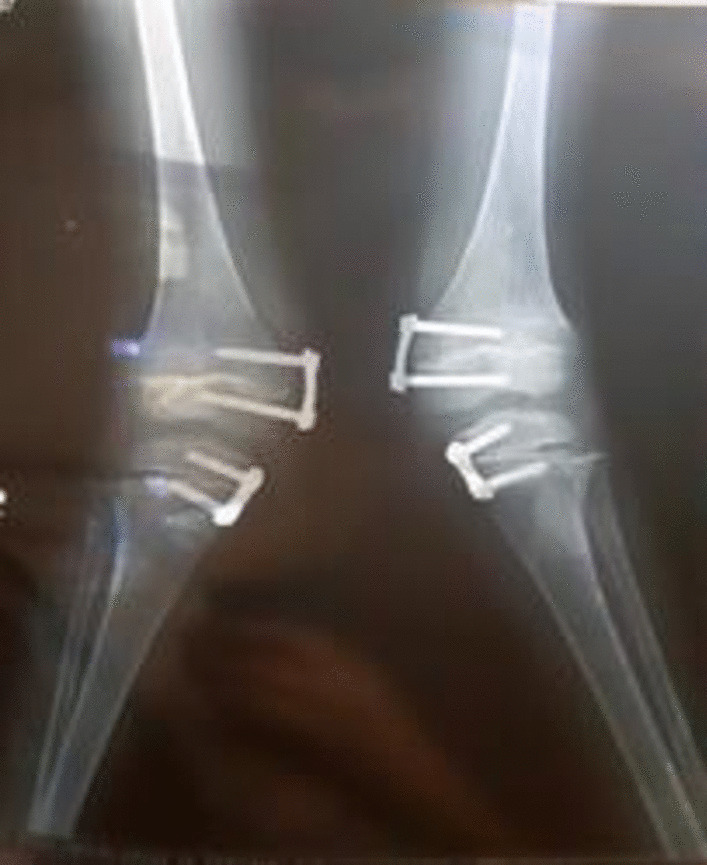
Postoperative radiograph following guided growth procedure showing improvement in the bilateral genu valgum deformity

At the age of 8 years and 9 months, following treatment for 9 months with potassium citrate, phosphorus, and vitamin D supplementation, the patient’s weight started to improve, increasing from 11 to 14 kg. Repeat phosphate levels showed a rise to 2.9 mg/dL. There was mild improvement in the patient’s genu valgum. Orthopedic surgery was consulted, and the patient underwent surgery for the correction of the genu valgum. Postoperative radiographs are shown in Fig. 3.

In addition, to the extensive laboratory workup done, genetic studies were sent out to evaluate for a genetic cause for her signs and symptoms. The result of next generation whole genome sequencing identified a new homozygous missense variant of ROGDI gene classified as a variant of unknown significance according to the American College of Medical Genetics.

**Fig. 4:**
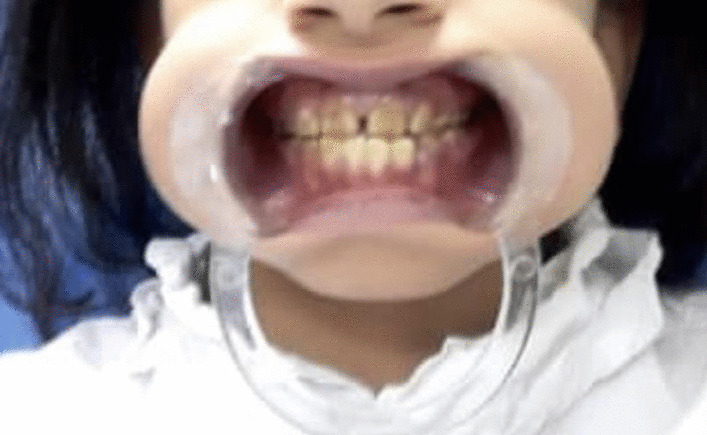
Patient’s teeth after undergoing multiple procedures for teeth alignment correction

A total of 18 months following the surgery there was a significant improvement in her gena valgus. The patient is now 10 years and 6 months of age, and her height is 116 cm which is in the 3rd percentile. She is still taking Polycitra-K 15 mL, phosphorus 10 mL three times daily, and alpha vitamin D 15 drops/day. Her weight is now up to 18 kg. The patient additionally underwent multiple procedures in attempt to correct her teeth malalignment with noticeable improvement as shown in Fig. 4.

## Discussion

The case presented here describes a child with significant metabolic bone disease, defined as those disorders of normal bone formation, remodeling, or mineralization. Common metabolic bone diseases include rickets, osteomalacia, and osteoporosis. Presentations suggestive of metabolic bone disease include lower extremity bowing, frontal bossing, growth restriction, widening of growth plates, and dental deformities. Metabolic bone disease is due to deficiency of calcium and/or phosphate; however, identifying the underlying cause of this deficiency can be challenging [6].

The first step in evaluating the cause of metabolic bone disease is a comprehensive evaluation, including history, physical examination, and laboratory work. A diagnosis of rickets is made using a combination of clinical, radiographic, and laboratory findings. This patient’s clinical presentation is suggestive of rickets, with a history of poor growth and lower extremity genu valgum. Laboratory findings and radiographic evidence further support a diagnosis of rickets, with increased alkaline phosphatase activity, decreased calcium, and normal parathyroid hormone (PTH) values. Radiographic findings were significant for mild osteopenic texture of visualized bones, bilateral lower extremity genu valgum, and a lucent fissure fracture line of the right fibula with evidence of healing attempts. Finally, the patient’s DEXA scan showed a *Z* score of −4 standard deviation (SD) below the mean, consistent with improper bone mineralization and supporting a diagnosis of rickets. Of note, genu valgum is commonly seen in children with rickets who develop structural weakness at an older age, as seen in our patient who presented at age 5 years [6, 11].

The causes of rickets are broad but can be quickly narrowed on the basis of laboratory findings. The hypophosphatemia seen here suggests hypophosphatemic rickets as the cause of this child’s metabolic bone disease. The causes of hypophosphatemia fall under three large categories: intracellular redistribution of phosphate, decreased intestinal absorption and increased renal excretion [12]. Intracellular redistribution can be due to increased insulin secretion, hungry bone syndrome, and acute respiratory alkalosis. Decreased intestinal absorption is seen in those with inadequate dietary phosphate intake, inhibition of intestinal absorption, steatorrhea and chronic diarrhea, and vitamin D deficiency or resistance. Finally, increased renal excretion of phosphate or phosphate wasting is associated with genetic causes of hypophosphatemia, renal tubular acidosis, Fanconi syndrome, and hyperparathyroidism [12].

Using the diagnostic algorithm put forth by Day *et al.* and Imel, the first step in approaching hypophosphatemia is evaluation of urinary phosphate [1, 13]. In patients with hypophosphatemia, the expected physiologic response would be increased renal reabsorption of phosphate and subsequently decreased urinary phosphate. The case presented here had a 24-hour urinary phosphate excretion of 362 mg, which is greater than expected in children with decreased serum phosphate [14]. This narrows the differential for our patient’s hypophosphatemia to those mechanisms causing increased renal excretion of phosphate; hyperparathyroidism, X-linked hypophosphatemia (XLH), renal tubular acidosis (RTAs), and Fanconi syndrome [1, 12, 13, 15, 16]. The patient’s parathyroid level was normal and genetic testing did not reveal evidence of XLH, making RTA or Fanconi syndrome more likely.

RTAs are a group of disorders of impaired excretion or reabsorption of H^+^ and HCO3 − in the renal tubules presenting with hyperchloremic normal anion gap metabolic acidosis, electrolyte derangements and a relatively preserved glomerular filtration rate [17]. There are four types of RTA, broadly broken down into those with hyperkalemia (RTA type 4 and voltage-dependent RTA) and those with hypokalemia (RTA type 1 and 2). This patient’s serum potassium was decreased, focusing the differential diagnosis on RTA type 1 (distal RTA) and RTA type 2 (proximal RTA). Distal RTA is due to impaired secretion of H*+* ions into the renal tubule at the distal convoluted tubule while proximal RTA is due to an inability to reabsorb secreted bicarbonate from the proximal renal tubule. An inability to reabsorb bicarbonate would lead to decreased serum bicarbonate levels and a compensated increase in serum chloride. Upon further laboratory workup, this patient was found to be mildly acidotic with a pH ranging from 7.27 to 7.34 (reference range 7.33–7.43), decreased serum bicarbonate (HCO_3_ −17, range 22–26 mmol/L), increased pCO_2_ 34 (range 23–29 mEq/L), decreased serum potassium 2.8 mmol/L (range 3.6–5.0 mmol/L) and increased chloride 112 mmol/L (range 96–106 mmol/L). These laboratory findings suggest a diagnosis of renal tubular acidosis. Diagnosing RTA with certainty is challenging in this case, as some laboratory workups were not completed, including those for urine calcium, urine anion gap, urine pH, HCO_3_^−^ loading test and NH_4_^+^ loading test [18, 19]. These values would have allowed for confirmation of a diagnosis of RTA and for differentiation between distal and proximal RTA.

There are reports of children with refractory rickets, similar to the case presented here, who were eventually diagnosed with renal tubular acidosis. A study by Joshi *et al.* conducted a retrospective chart review of 36 cases of refractory rickets in children ages 1.5 months to 13 years of age. Refractory rickets was defined as a lack of response to known therapeutic doses of vitamin D. The authors found that the 23 cases met a diagnosis of RTA, 20 of which were distal RTA and 3 of which were proximal RTA [18]. Clinical presentation of those with RTA included polyuria, decreased growth and failure to thrive, with features of rickets including costochondral beading, wrist widening, and bowing of the legs.

The patient’s genetic testing raised an interesting new possibility in the ultimate diagnosis of this patient. The VUS identified in this patient is a mutation in the ROGDI gene. Biallelic mutations in the ROGDI gene are associated with Kohlschutter-Tönz syndrome (OMIM# 226750), which is characterized by global developmental delay, early-onset intractable seizures, spasticity, and amelogenesis imperfecta affecting both primary and secondary teeth and causing yellow or brown discoloration of the teeth.

The variant identified here is not found in public genetic databases, affects a highly conserved amino acid residue, and is predicted by various bioinformatic tools to be deleterious. As the variant identified in this case is a missense and all reported cases in the literature are loss of function variants, we believe that missense variants in this gene may be associated with a variant of the disorder involving possible amelogenesis imperfecta. Amelogenesis imperfecta would explain this patient’s dental findings.

Given that the amino acids affected by this mutation are proposed to either be more widely expressed in extradental tissues than previously thought or cause an underlying interstitial matrix abnormality, the mutation could explain the patient’s nephrogenesis and RTA, ultimately explaining the child’s hypophosphatemic rickets and physical exam findings [10].

## Conclusion

Metabolic bone disease is diagnostically challenging in that the differential diagnosis is broad and requires a comprehensive history, physical exam, and laboratory workup. Our diagnostic workup was additionally complicated by constraints in the laboratory evaluations available in Yemen and Egypt. There is therefore some limitation in our ability to make a concrete final diagnosis; however evidence thus far supports a diagnosis of AI and RTA causing hypophosphatemic rickets. Genetic testing identified a new missense mutation in the ROGDI gene; deleterious mutations of which have been associated with AI and Kohlschutter-Tönz syndrome. Further research is required to determine whether this patient’s underlying genetic abnormality explains both the AI and renal findings seen in this case; however, given existing theories in this literature as to the more widely expressed dental proteins or variations in interstitial matrix, this may be a possibility. Through this case we provide an approach to metabolic bone disease workup, as well as a new VUS that may explain a challenging rickets case to diagnose and treat.

## Data Availability

All data are included in the medical record of the patient. Clinical data are available from the corresponding author but only on reasonable request.

## Abbreviations

VUS: Variant of unknown significance
AI: Amelogenesis imperfecta
CRP: C-reactive protein
GH: Growth hormone
ILGF-1: Insulin-like growth factor 1
PTH: Parathyroid hormone
XLH: X-linked hypophosphatemia
RTA: Renal tubular acidosis
